# Is prehospital intubation of severely injured children in accordance with guidelines?

**DOI:** 10.1186/s12873-022-00750-1

**Published:** 2022-12-06

**Authors:** Teresa Maek, Ulrike Fochtmann, Anna von Loewenich, Pascal Jungbluth, Werner Zimmermann, Rolf Lefering, Sven Lendemans, Bjoern Hussmann

**Affiliations:** 1 Department of Orthopedics and Trauma Surgery, Alfried Krupp Hospital Essen, Alfried-Krupp-Straße 21, 45131 Essen, Germany; 2 Department of Pediatrics 1, University Hospital Essen, Hufelandstraße 55, 45122 Essen, Germany; 3 Department of Orthopedics and Trauma Surgery, University Hospital Duesseldorf, Moorenstraße 5, 40225 Duesseldorf, Germany; 4 Institute for Research in Operative Medicine (IFOM), Witten/Herdecke University, Ostmerheimer Straße 200, 51109 Cologne, Germany; 5 University of Duisburg-Essen, Hufelandstraße 55, 45122 Essen, Germany

**Keywords:** Trauma registry, Multivariate regression analysis, Prehospital intubation severely injured children, Outcome

## Abstract

**Background:**

The current German S3 guideline for polytrauma lists five criteria for prehospital intubation: apnea, severe traumatic brain injury (GCS ≤8), severe chest trauma with respiratory failure, hypoxia, and persistent hemodynamic instability. These guideline criteria, used in adults in daily practice, have not been previously studied in a collection of severely injured children. The aim of this study was to assess the extent to which the criteria are implemented in clinical practice using a multivariate risk analysis of severely injured children.

**Methods:**

Data of 289,698 patients from the TraumaRegister DGU® were analyzed. Children meeting the following criteria were included: Maximum Abbreviated Injury Scale 3+, primary admission, German-speaking countries, years 2008–2017, and declaration of intubation. Since children show age-dependent deviating physiology, four age groups were defined (years old: 0–2; 3–6; 7–11; 12–15). An adult collective served as a control group (age: 20–50). After a descriptive analysis in the first step, factors leading to prehospital intubation in severely injured children were analyzed with a multivariate regression analysis.

**Results:**

A total of 4489 children met the inclusion criteria. In this cohort, young children up to 2 years old had the significantly highest injury severity (Injury Severity Score: 21; *p* ≤ 0.001). Falls from both high (> 3 m) and low heights (< 3 m) were more common in children than in adults. The same finding applied to the occurrence of severe traumatic brain injury. When at least one intubation criterion was formally present, the group up to 6 years old was least likely to actually be intubated (61.4%; *p* ≤ 0.001). Multivariate regression analysis showed that Glasgow Coma Scale score ≤ 8 in particular had the greatest influence on intubation (odds ratio: 26.9; *p* ≤ 0.001).

**Conclusions:**

The data presented here show for the first time that the existing criteria in the guideline for prehospital intubation are applied in clinical practice (approximately 70% of cases), compared to adults, in the vast majority of injured children. Although severely injured children still represent a minority of all injured patients, future guidelines should focus more on them and address them in a specialized manner.

## Background

Severely injured children up to 16 years of age are still a rarity in Germany. This fact was also shown by the current TraumaRegister DGU® annual report from 2021, in which only 3.5% of more than 28,000 documented severely injured patients were children [1]. Nevertheless, the consequence of severe injury in this age group remains the leading cause of death worldwide [2]. It is due to this low incidence that scientific analysis is fundamentally difficult and even more so with a high level of evidence. This limitation is increasingly evident, especially in the prehospital setting.

Epidemiological studies have shown that acute neurological disorders, acute respiratory disorders, and trauma are the most common prehospital interventions in children [3, 4]. A combination of severe trauma and its consequence of acute respiratory distress are known to be one reason for hypoxia and resulting cardiac arrest, especially in young children with physiologically lower oxygen reserves [5]. Therefore, due to Advanced Trauma Life Support (ATLS®), respiration should be evaluated in a targeted manner and the child treated if necessary [6]. In this context, further damage after trauma must be avoided at all times (“Do not further harm”) [6]. Therefore, the current European Resuscitation Council (ERC) guideline does not exclusively list tracheal intubation as a treatment option, especially in children, but also mentions supraglottic ventilation as an alternative, especially since a team trained in tracheal intubation should perform it in children [5].

Despite the high importance of respiration and ventilation for severely injured children, there are no recommendations explicitly for children in the current German S3 guideline on polytrauma [7]. General information, e.g., about the indications for prehospital intubation, is provided. However, this information mainly refers to adult patients. Previous studies by our research group tended to show a transfer of findings to the child with regard to prehospital volume therapy and its consequences, but similar results do not yet exist for prehospital intubation [8]. The current German guidelines list five indications for intubation at the scene of an accident: apnea, severe chest trauma with respiratory insufficiency, hypoxia (SpO^2^ < 90%), severe traumatic brain injury (TBI) with a Glasgow Coma Scale (GCS) score ≤ 8, and persistent hemodynamic instability with a systolic blood pressure < 90 mmHg [7]. Although these criteria initially appear logical and transferable, no scientific evidence specific to severely injured children exists in the guideline, as previously mentioned. The S2k guideline on polytrauma in children and adolescents also specifies intubation criteria. These almost correspond to the above-mentioned criteria of the S3 guideline Polytrauma: apnea or gasping (respiratory rate < 6), hypoxia with SpO2 < 90%, severe traumatic brain injury, hemodynamic instability, severe chest trauma with respiratory instability and hypoventilation [9].

A general recommendation for prehospital ventilation in children is provided by the currently published S1 guideline for prehospital airway management [10]. However, this guideline not only refers to children after trauma but also to pediatric emergency medicine as a whole. The indications for ventilation are basically comparable with the German S3 guideline for severely injured patients. However, this guideline points out that endotracheal intubation should only be used when all other therapies to restore or maintain normoxia have been unsuccessful, since in children in particular, optemized mask ventilation or a supraglottic airway can be equally successful [10]. Especially since intubation in children requires an experienced team and seems to be more prone to complications [11]. In their study, Lockey et al. described more frequent incorrect intubation into the esophagus or right main bronchus, for example [12]. Similarly, multiple intubation attempts are often required until achieving successful endotracheal intubation [13]. Last but not least, studies have described injury and “swelling” of the airway after frustrated attempts, especially in infantile soft and significantly more responsive airways. DiRusso et al. even concluded in their study that prehospital intubation is an independent risk factor for worse outcomes [14]. Similarly, Schauer et al. showed in their study of patients from Iraq and Afghanistan that children intubated at the scene of an accident had a worse outcome than children intubated in the hospital [15]. However, children at accident scenes had higher overall injury severity.

A recent multivariate study by Hawkins et al. examined independent predictors of increased mortality [16]. Here, age, total injury severity, and neurologic parameters were found to strongly influence mortality. However, the site of intubation (accident site or hospital) itself did not [16]. Nevin et al. also concluded in their analysis that accident scene intubation is a safe procedure for children [17]. With regard to this study, it must be pointed out that these severely injured patients were treated by a highly specialized and trained emergency team with a high patient volume. These results are not generally transferable. Especially in children, other authors in the current literature still consider this prehospital measure as a high risk intervention [18–20].

Due to the described discrepancy of conclusions in the literature and the guidelines with little focus on severely injured children, the fundamental question arises of whether children are treated differently after severe trauma in the field of airway management compared to comparably injured adult patients. Therefore, the aim of the present study was to investigate whether severely injured children are treated similarly to the German S3 polytrauma guidelines. The focus of the study was to examine the intubation criteria of the German S3 polytrauma guideline for severely injured patients as described above. Similarly, the outcome of the severely injured children in the absence and presence of intubation was investigated in this analysis.

## Methods

The TraumaRegister DGU® of the German Trauma Society (Deutsche Gesellschaft für Unfallchirurgie, DGU) was founded in 1993. The aim of this multicenter database is to provide pseudonymous and standardized documentation of severely injured patients.

The data were collected prospectively in the following four consecutive time phases from the site of the accident until discharge from the hospital: A) prehospital phase; B) emergency room and initial surgery; C) intensive care unit (ICU); and D) discharge. The documentation includes detailed information about demographics, injury pattern, comorbidities, pre- and in-hospital management, progression in the intensive care unit, and relevant laboratory findings, including data on transfusion and the outcome of each individual patient. The inclusion criterion was hospital admission via the emergency room with subsequent ICU or hospital arrival with vital signs and death before admission to the ICU. The infrastructure for documentation, data management, and data analysis is provided by the AUC - Academy for Trauma Surgery (AUC - Akademie der Unfallchirurgie GmbH), a company affiliated with the German Trauma Society. The scientific leadership is provided by the Committee on Emergency Medicine, Intensive Care and Trauma Management (Sektion NIS) of the German Trauma Society. The participating hospitals submit their pseudonymized data to a central database via a web-based application. The scientific data analysis is approved according to a peer review procedure established by Sektion NIS. The participating hospitals (90%) are primarily located in Germany; however, an increasing number of hospitals from other countries (such as Austria, Belgium, China, Finland, Luxembourg, Slovenia, Switzerland, the Netherlands, and the United Arab Emirates) also contribute data. Currently, the data for more than 35,000 patients from almost 700 hospitals have been entered into the database annually. Participation in the TraumaRegister DGU® is voluntary. For hospitals associated with the TraumaNetzwerk DGU®, however, the entry of at least one basic dataset is obligatory for quality assurance reasons (this section of the method part was previously published in [21, 22]). The present study was consistent with the publication guidelines of the TraumaRegister DGU® (TR-DGU) and was registered under the TR-DGU project ID 2018–046. Full approval was obtained from the Ethics Committee of the Medical Association of North Rhine (internal number: 165/2022).

Only patients from participating hospitals in Germany, Austria and Switzerland were included in the study to ensure comparability.

Sepsis was defined according to the American College of Chest Physicians/Society of Critical Care Medicine (ACCP-SCCM) consensus conference [23]. Organ failure was classified according to a Sequential Organ Failure Assessment (SOFA) score ≥ 3 [24]. Hospitals participating in the TR-DGU enter the SOFA score as an overall value. Therefore, no conclusions can be drawn regarding individual patient therapy or interventions. Multiorgan failure (MOF) was included in the analysis if at least two independently affected organ systems had pathology.

Children documented in the TR-DGU between 2008 and 2017 who met the following inclusion criteria were analyzed:

1. primary admitted patients (not transferred out within 48 h after admission);
2. Maximum Abbreviated Injury Scale (MAIS) score of 3+;
3. information about prehospital intubation (yes/no) available; and
4. treated in a German, Austrian, or Swiss hospital.

Since children have different physiological parameters due to their ages, four age groups were defined:

1. *0–2 years old (n = 575; infant);*
2. *3–6 years old (n = 803; toddler);*
3. *7–11 years old (n = 1341; school-age child); and*
4. *12–15 years old (n = 1970; adolescent).*

Corresponding to this group classification, different physiological normal values were obtained with respect to respiratory rate and systolic blood pressure compared to adults:

Respiratory rate (modified from [25]):

1. Infant: 20–40 per minute;
2. Toddler: 20–30 per minute;
3. School-age child: 16–24 per minute; and
4. Adolescent: 12–20 per minute.

Systolic blood pressure (modified from [25]):

1. Infant: 80–96 mmHg;
2. Toddler: 95–98 mmHg;
3. School-age child: 97–106 mmHg; and
4. Adolescent: 106–114 mmHg.

**Fig. 1:**
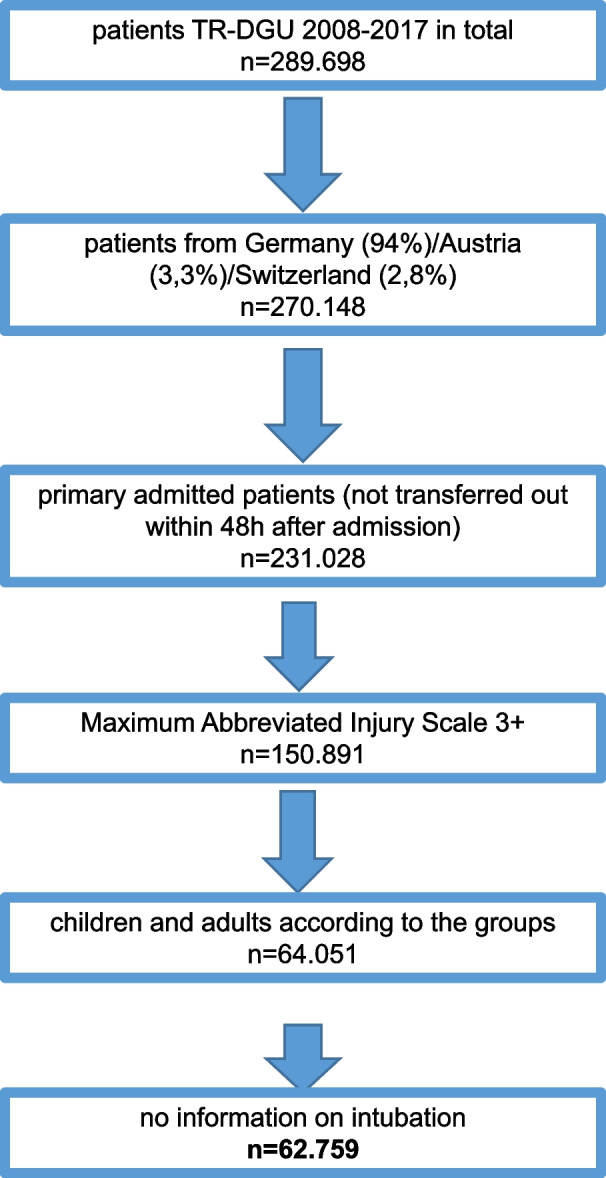
Graphic representation of the test procedure

An adult collective (age 20–50 years; *n* = 58,070) served as the control group. A total of 4689 patients fulfilled the inclusion criteria and could be analyzed (Fig. 1).

Further investigation was conducted in three steps.

1. Descriptive univariate analysis was performed considering age groups. Among others, the following outcome-influencing parameters of the patients were examined: overall injury severity according to the Injury Severity Score (ISS), New Injury Severity Score (NISS), prehospital parameters such as heart rate, blood pressure, Glasgow Coma Scale (GCS) score, and hospital and ICU length of stay.
2. Also in a univariate analysis, four intubation criteria from the current S3 guideline were examined on an age group-specific basis (severe TBI with a GCS ≤8; hereafter referred to as “unconsciousness”, persistent hemodynamic instability [systolic blood pressure < 90 mmHg]; hereafter referred to as “shock”, apnea or hypoxia [SpO_2_ < 90%]; hereafter referred to as “low oxygen saturation“, severe chest trauma with respiratory failure [respiratory rate >29/min., in children younger than 6 years old, respiratory rate <10 or >39/min.]; hereafter referred to as “respiratory failure”). The fifth intubation criterion “apnea or gasping (respiratory rate<6)” was not checked since it must be assumed that intubation must always be performed in these cases without exception. The adaptation of the intubation criteria to the child’s physiology, as recommended in the S3 guideline, was conducted in particular for children younger than 6 years of age since the physiological difference from older children is greatest here. Due to the lack of controlled studies, to the authors’ knowledge, no clear guideline values exist for intubation in severely injured children [26]. Therefore, for the analysis of the intubation criterion of “respiratory failure” in children younger than 6 years old, not only hyperventilation with > 39/min. But also hypoventilation with < 10/min. With simultaneous chest trauma was included. Following a pathological respiratory rate > 29/min. in adults (normal value adults 12–20/min.), the analysis in children younger than 6 years old was performed at a respiratory rate > 39/min. to investigate pathological values with simultaneous chest trauma as reliably as possible. This procedure is in accordance with the S2k guideline recommendation “Polytrauma care in childhood” [9]. The same applies to the review of shock as an intubation criterion. Here, also due to other physiological norm values, especially in infants from Group 1, systolic blood pressure < 90 mmHg as a shock parameter must be critically discussed. However, since systolic blood pressure values
  i. > 90 mmHg can also be normal in this group, this intubation criterion was also examined in Group 1.
  ii. First, we examined how many patients met at least one criterion in each group and, further, whether intubation actually occurred when an intubation criterion was met. In addition, the actual mortality in the presence of at least one intubation criterion, compared with the statistically expected mortality, was analyzed between the groups. RISC II (Revised Injury Severity Classification) was applied for prognosis [27].
3. In the last step, multivariate logistic regression analysis with “prehospital intubation” as the dependent variable was used to investigate the influences of early findings. The analysis was performed in both collectives of adult and pediatric patients. The following 12 predictors were included in the analysis: GCS ≤ 8; systolic blood pressure < 90 mmHg; peripheral saturation SpO_2_ < 90%; severe chest trauma with an Abbreviated Injury Scale [AIS] ≥3 and respiratory rate > 29; children up to 6 years of age respiratory rate < 10 or > 39; mode of transport, relevant injury (AIS ≥ 3) of the head, thorax, abdomen, and extremities; accident year; and ISS ≥ 16. These predictors do not represent absolute intubation criteria.

### Statistics

Data were analyzed using Statistical Package for the Social Sciences software (SPSS®; version 25, IBM Inc., Armonk, NY, USA). Formal statistical testing of differences in groups was not performed because the very large number of cases would render even small and clinically irrelevant differences statistically significant. Furthermore, four children subgroups plus an adult control group would induce a large number of pairwise test results per variable tested. Continuous variables are presented as the mean ± standard deviation, and incidences are presented as percentages. The results of multivariate logistic regression analysis are presented as odds ratios (ORs) with 95% confidence intervals.

## Results

### Descriptive analysis

Descriptive analyses showed that the majority of children and adults involved in accidents were male (Table 1). However, in the group of infants, this effect was less prominent (59.2%). Most children suffered blunt trauma (94.3%). In terms of injury severity according to the ISS and NISS, the results were similar (Table 1). Here, Group 1 initially had higher ISS and NISS on average than Groups 2 and 3. This finding was similar when considering severe head injuries with an AIS ≥3. Again, of all groups, group 1 of infants was the highest. When analyzing the causes of accidents, Group 1 had the greatest proportion of patients with a fall (low falls from < 3 m and high falls > 3 m) (Table 2). In contrast, accidents while riding bicycles in particular were most common in Group 4 (adolescents).

**Table 1:** Descriptive data from severely injured children compared with adults

Group	Infant	Toddler	School-age child	Adolescent	Adults
Male sex (%)	59.2	61.7	63.9	63.0	77.7
Age in years (MV, SD)	1.1 ± 0.8	4.6 ± 1.1	9.1 ± 1.4	13.8 ± 1.1	35.4 ± 9.6
ISS (MV, SD)	20.5 ± 13.3	19.4 ± 12.5	18.5 ± 10.6	20.6 ± 12	21.9 ± 12.2
NISS (MV, SD)	27.1 ± 17.2	24.5 ± 14.9	23.1 ± 13.4	25.5 ± 14.3	27 ± 14.2
Blunt trauma (%)	97.3	97.0	97.8	96.1	94.1
AIS head ≥3 (%)	61.9	58.7	50.8	45.8	36.0
AIS thorax ≥3 (%)	27.7	27.5	24.5	34.6	52.1
AIS abdomen ≥3 (%)	5.0	9.5	14.1	17.8	16.7
AIS extremities including pelvis ≥3 (%)	18.1	31.1	36.6	35.1	36.8
AIS face ≥3 (%)	8.5	10.3	9.7	10.9	12.4

Values are the mean (MV), standard deviation (SD) or % of the group; *ISS* Injury Severity Score, *NISS* New Injury Severity Score, *AIS* Abbreviated Injury Scale

**Table 2:** Causes of accidents and accident history in group-specific comparison

Group	Infant	Toddler	School-age child	Adolescent	Adults
Any Traffic accident (%)	31.4	48.2	66.2	66.4	61.3
Traffic accident, automobile (%)	14.7	12.7	11.8	13.9	28.4
Traffic accident, motorbike (%)	0.2	0.3	1.5	13.0	19.0
Traffic accident, bicycle (%)	2.7	6.8	17.3	20.5	7.0
Traffic accident, pedestrian (%)	12.7	27.0	33.5	16.1	5.3
Fall ≥3 m (%)	26.9	22.4	13.8	15.1	18.0
Fall < 3 m (%)	23.2	14.4	11.0	8.7	8.6
Other accidents (%)	19.6	16.5	11.0	12.8	13.6

Values are the mean, standard deviation (SD) or % of the group

**Fig. 2:**
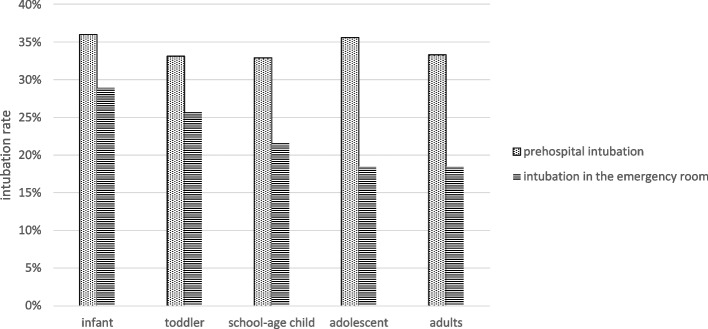
Frequency of intubation in the different groups

**Fig. 3:**
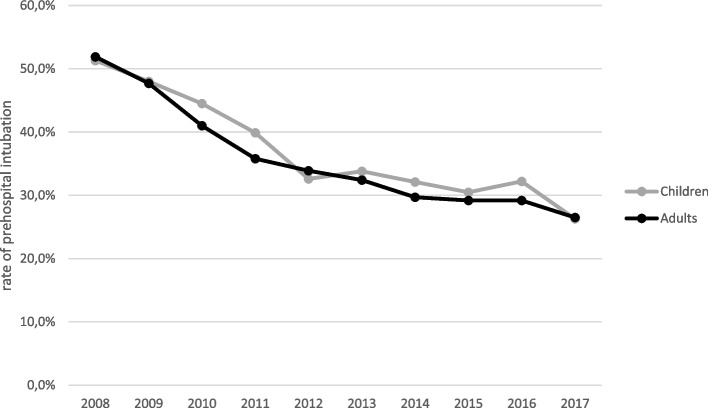
Frequency of intubation in the studied cohort of severely injured children and adults from 2008 to 2017

As shown in Table 3, prehospital measurements at the site of the accident were similarly distributed across all groups. However, resuscitation at the scene and prehospital administration of catecholamines on admission to the hospital were highest in Group 1. The older that the children became, the more likely that they were to use an alternative airway. However, this intervention remained a rarity overall, with a rate of 2% (of which adults accounted for the largest proportion) (Table 3). Figure 2 shows the rate of intubation in the different groups. Both prehospital intubation and intubation in the emergency room were compared (Fig. 2). As shown in Fig. 3, in the studied collective of severely injured children, the frequency of intubations has decreased overall but has remained stable at similar levels since 2012.

**Table 3:** Group-specific clinical data from prehospital treatment and emergency room therapy

Group	Infant	Toddler	School-age child	Adolescent	Adults
BP at accident site (mm Hg; MV, SD)	105 ± 40	105 ± 29	111 ± 25	117 ± 27	123 ± 31
Heart rate at accident site (sec; MV, SD)	112 ± 39	108 ± 28	101 ± 25	96 ± 25	93 ± 24
Respiratory rate at the accident site (MV, SD)	19 ± 9	19 ± 7	17 ± 7	16 ± 7	16 ± 6
GCS at the accident site	10.6 ± 4.8	11.5 ± 4.4	11.9 ± 4.4	11.7 ± 4.4	12.2 ± 4.3
Fluid volume replaced prehospital (%)	69.6	80.2	85.3	88.6	89.5
Fluid volume replaced prehospital (ml; MV, SD)	293 ± 268	398 ± 294	549 ± 386	799 ± 622	928 ± 694
Fluid volume replaced in the emergency department (ml; MV, SD)	355 ± 325	545 ± 550	728 ± 809	1194 ± 1350	1490.8 ± 1771.5
Prehospital use of catecholamines (%)	10.0	8.2	6.6	8.7	9.1
Prehospital sedation (%)	55.1	65.3	68.6	72.9	74.3
Prehospital chest tube (%)	2.6	1.7	0.6	3.0	4.5
Prehospital CPR (%)	10.4	6.5	4.0	4.2	3.5
Prehospital alternative airway (%)	0.6	0.8	0.0	0.9	2.1
BP < 90 mmHg in the emergency department (%)	30.8	20.1	11.1	8.2	10.2

*BP* Blood pressure, Values are the mean, standard deviation (SD) or % of the group, *GCS* Glasgow Coma Scale, *CPR* Cardiopulmonary resuscitation

For the outcomes of hospital length of stay, level of care provided by the treating hospital, and outcomes from Table 4, the results were again similarly distributed across groups. However, mortality was highest in Group 1. Among the groups, 33.0% of intubated children with a GCS score < 9 died (Table 5).

**Table 4:** Group-specific outcomes of severely injured children compared with adults

Group	Infant	Toddler	School-age child	Adolescent	Adults
Helicopter transport (%)	30.9	34.4	34.6	31.6	28.4
Level of the hospital (%)					
Level 1	77.2	80.0	74.6	69.2	68.2
Level 2	18.3	16.6	21.1	24.7	24.7
Level 3	4.5	3.5	4.3	6.1	7.1
Total prehospital time (minutes; MV, SD)	61.0 ± 30.2	59.7 ± 25.7	60.2 ± 25.5	61.9 ± 26.3	64.0 ± 27.4
Days in the intensive care unit (MV, SD)	6.3 ± 10.5	4.8 ± 7.5	4.6 ± 7.6	5.8 ± 9.2	7.1 ± 10.7
Days intubated (MV, SD)	2.6 ± 6.0	1.8 ± 4.7	1.8 ± 4.5	2.3 ± 5.9	3.4 ± 7.8
Days of hospitalization (MV, SD)	13.9 ± 15.7	11.8 ± 10.8	12.7 ± 11.0	15.4 ± 16.0	19.3 ± 19.9
Expected mortality based on RISC II (MV)	11.9 ± 26.4	7.2 ± 20.8	6.8 ± 19.3	8.6 ± 21.1	8.0 ± 20.0
Died in hospital (%)	15.7	8.3	6.3	7.2	7.8

Values are the mean, standard deviation (SD) or % of the group, *RISC* Revised Injury Severity Classification, *ICU* Intensive care unit

**Table 5:** Expected risk of death (based on RISC II) and observed hospital mortality in intubated children and adults with a prehospital Glasgow Coma Scale score < 9

	Children intubated (***n*** = 1003)	Adults intubated (n = 10,119)
Expected mortality based on RISC II (%)	32.1	33.3
Hospital mortality (95% CI)	33.0 (30.1–35.9)	33.6 (32.7–34.9)

### Analysis of intubation criteria according to the German S3 polytrauma guideline

**Fig. 4:**
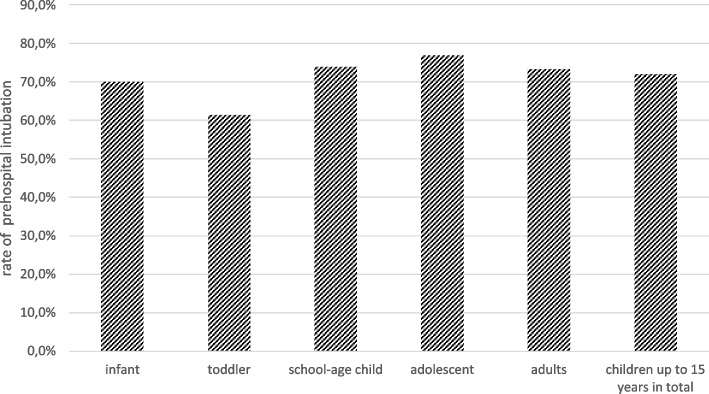
Rate of prehospital intubation when at least one intubation criterion is met according to the S3 guideline Polytrauma

Table 6 presents the percentage of intubation criteria according to the German S3 polytrauma guidelines. Overall, it was shown that the highest parameters for intubation existed, especially in Groups 1 and 2. The same was true for the overall analysis if at least one intubation criterion was present (Group 1: 43.7%, Group 4: 32.0%; *p* < 0.001). Figure 4 shows the actual frequency of intubations in the groups when at least one intubation criterion was present.

**Table 6:** Presence of the four prehospital intubation criteria according to polytrauma guidelines

Group	Infant	Toddler	School-age child	Adolescent	Adults
Unconsciousness (%)	33.3	25.7	23.1	25.0	20.6
Shock (%)	30.3	26.8	16.0	11.9	12.7
Low oxygen saturation (%)	12.3	6.9	6.1	10.5	13.2
Respiratory failure (%)	12.0	4.9	5.3	4.2	2.9
at least 1 criterion for intubation met (%)	43.7	39.8	31.5	32.0	29.4

The mortality of the total group of severely injured patients was highest in the “intubated” group in both children and adults (Table 7). When at least one intubation criterion was present, the result was almost the same. However, mortality was higher in this group (Table 7). The prognosis of death, measured by RISC II, corresponded well with the actual mortality (children up to 15 years old intubated with one intubation criterion: RISCII 29.2, children up to 15 years old’s actual mortality 29.9%; *p* < 0.001).

**Table 7:** Mortality in prehospital nonintubated and intubated children compared with adults as a whole group and in the presence of at least one intubation criterion

Patient group		Children	Adults
Not intubated	Number of patients	*n* = 3074	*n* = 38,757
	Expected mortality (%)	1.3	1.8
	Observed mortality (%, 95 CI, n)	1.1 [0.7–1.5] *n* = 34	1.4 [1.3–1.5] *n* = 531
Intubated	Number of patients	*n* = 1615	n = 19,313
	Expected mortality (%)	21.5	20.5
	Observed mortality (%, 95 CI, n)	21.7 [19.7–23.7] *n* = 350	20.6 [20.0–21.6] *n* = 3979
Not intubated, but at least one criterion for intubation met	Number of patients	*n* = 440	*n* = 4468
	Expected mortality (%)	4.1	6.5
	Observed mortality (%, 95 CI, n)	4.1 [2.2–5.9] *n* = 18	6.6 [5.9–7.4] *n* = 297
Intubated, and at least one criterion for intubation met	Number of patients	*n* = 1134	*n* = 12,262
	Expected mortality (%)	29.2	29.5
	Observed mortality (%, 95 CI, n)	29.9 [27.2–32.6] *n* = 339	29.7 [28.9–30.5] *n* = 3644

Expected mortality based on RISC II, Revised Injury Severity Classification version II

### Multivariate regression analysis regarding the dependent risk characteristic “prehospital intubation” (*n* = 1418)

*A* GCS score < 9 had the highest influence on prehospital intubation among all parameters in children (odds ratio 26.6, 95% CI: 17.5–41.3; *p* < 0.001) (Table 8). The presence of prehospital shock (RRsys < 90 mmHg) and a pulse oximetry-measured peripheral SpO_2_ < 90% were also significantly more likely to result in intubation. Of the intubation criteria listed in the S3 guideline, only decreased or highest increased respiratory rate with concomitant severe chest trauma showed a nonsignificant outcome for prehospital intubation (Table 8). Injury severity with an ISS ≥ 16 also showed no significant effect. Among injury severities, severe head injury with an AIS ≥ 3 significantly represented a predictor of intubation.

**Table 8:** Multivariate regression analysis with prehospital intubation as the dependent variable in Children and adults

	Children (*n* = 1418)			Adults (*n* = 17,744)		
	Odds Ratio	95% CI	***p*** values	Odds ratio	95% CI	***p*** values
Unconsciousness	26.9	17.5–41.3	< 0.001	22.8	20.0–26.1	< 0.001
Shock	1.6	1.1–2.5	0.02	2.2	1.9–2.5	< 0.001
Low oxygen saturation	3.2	1.6–6.5	< 0.001	2.8	2.4–3.1	< 0.001
Respiratory failure	2.0	0.8–4.9	0.1	2.2	1.6–2.9	< 0.001
Helicopter transport	3.5	2.5–4.8	< 0.001	3.1	2.8–3.3	< 0.001
AIS face ≥2	2.2	1.4–3.5	< 0.001	1.6	1.4–1.8	< 0.001
AIS head ≥3	3.2	2.1–5.0	< 0.001	1.6	2.1–5.0	< 0.001
AIS thorax ≥3	2.8	1.9–4.1	< 0.001	1.3	1.1–1.4	< 0.001
AIS abdomen ≥3	1.0	0.6–1.8	0.9	0.8	0.8–1.0	0.02
AIS extremities/pelvis ≥3	1.8	1.2–2.8	0.004	1.8	1.6–2.0	< 0.001
ISS ≥ 16	1.2	0.8–1.8	0.28	1.5	1.4–1.7	< 0.001

*AIS* Abbreviated Injury Scale, *ISS* Injury Severity Score

A comparable result was seen in the severely injured adults (Table 8). Here, however, all intubation criteria according to the German S3 polytrauma guideline were significant predictors of prehospital intubation. Unlike in children, total injury severity and injury severity with an ISS ≥16 also had highly significant influences on intubation (ISS ≥16: odds ratio 1.5, 95% CI: 1.4–1.7; *p* < 0.001).

## Discussion

In the present study, it was shown for the first time on the basis of a large patient cohort of severely injured children that the criteria from the German S3 polytrauma guidelines for prehospital intubation can also be applied to children. Thus, a general transferability from adults to children seems to be possible in principle. However, it must always be kept in mind that intubation per se is not the goal-directed measure, but is a possible form of therapy for the restoration of normoxia, normoventalation and normotension.

Examining the descriptive data, it is not surprising that the majority of severely injured children are male. This finding reflects the previous data situation regarding severely injured patients in the TR-DGU and the current literature [1]. Thus, Ilie et al. came to a comparable conclusion in their study [28]. Interestingly, however, the distribution of gender in children, especially the younger that they are, is somewhat more balanced than that in the adult collective. In addition, blunt trauma represents the largest proportion, with a mean greater than 94.3%, as would be expected. This injury remains the leading injury entity in German-speaking countries and in Europe as a whole. Injury severity measured with the ISS or NISS showed little variation between groups. However, infants up to 2 years of age were more severely injured and more comparable to the adult group. In a retrospective registry study, it remained speculative, of course, why the infants in particular appeared to be more severely injured. Here, one explanation could lie in the causes of accidents. Due to falls, both from low (< 3 m) and very high (> 3 m) heights, there was a high prevalence of falls in Group 1. Correspondingly, severe head injuries with an AIS ≥3 in comparison also occurred most frequently in this group. In contrast, both in the current literature and in the present study, traffic accidents and their consequences (e.g., increased severe limb injuries with an AIS of ≥3) increase with increasing age of children [29, 30].

The association between severe head injuries and very young children has been well described in the current literature. For example, Haarbauer-Krupa et al. showed in their study, analogous to the present results, that falls are the leading cause of accidents in children up to 4 years of age and, in this context, severe TBI occurs disproportionately [31]. Prehospital measures also emphasize these findings. Thus, it seems initially conclusive that prehospital resuscitation, prehospital administration of catecholamines, and initial lower GCS scores were also most common in the group of youngest children, in contrast to the other groups. Interestingly, prehospital rescue time was shorter in children than in adults. This outcome was also demonstrated by Ashburn et al. in their study [32]. One reason could be the cause of the accident itself. As already mentioned, falls in particular represent a leading cause, so potentially complex rescues of trapped patients, e.g., from cars, occurred less often.

It is clear from both the present study and the current literature that severe TBI in particular is by far the most common cause of death in children [33]. Accordingly, in this study, the proportion of children dying with an initial GCS score < 9 was more than 30%. Thus, based on these results, as well as a review of current studies, it is completely indisputable that a major focus in the treatment of severely injured children must be the prehospital securing of the airway and breathing or ventilation. In this context, prehospital securing of the airway and maintenance of at least adequate oxygenation play crucial roles due to severe TBI [34]. These roles are illustrated not least by the high prioritization of the airway in internationally recognized treatment concepts, such as the ATLS® [6].

Therefore, the most remarkable finding of this study is that the intubation criteria listed in the German S3 polytrauma guideline are apparently also applied in practice in pediatric patients. Approximately 30% of all patients fulfilled at least one intubation criterion, and of these patients, more than 70% were actually intubated, both adults and children. Transferability of the guideline from the adult to the severely injured child thus seems to have been actually applied in practice. It should be mentioned once again that absolute causality cannot be readily deduced on the basis of retrospective registry data. Thus, it remains unclear why intubation was not performed in the remaining 30% of severely injured children despite an intubation criterion. It must be noted here that the prehospital decision for or against an intervention, e.g., intubation, must be made individually by the acting emergency team and cannot be conclusively clarified in a retrospective analysis with pseudonymized data. Previous studies have shown that intubation is also associated with risks and can negatively influence the outcome if the indication is not clear [35]. When examining the results of the adult cohort, it is also notable that the prehospital decision for intubation was also not made in approximately 30%, although an intubation criterion according to the S3 polytrauma guideline existed in these cases as well. Thus, there were no statistically significant differences and in practice between the adult and pediatric populations in this regard. Similar results were obtained by Laurer et al. in their study [29]. With the help of a matched pairs analysis, they were also able to show that the prehospital treatment of severely injured children is comparable in principle to that of adults and that there are only differences in the causes of accidents and previous illnesses.

Interestingly, it could be shown that children who were not intubated, despite an existing intubation criterion, died in only approximately 4.1% of cases. Thus, the proportion was significantly lower than in adults, with 6.6% of nonintubated casualties having an existing intubation criterion. Almost 30% who met an intubation criterion and were intubated died. This finding suggests that children with an existing intubation criterion might have been more severely injured, but the prehospital individual decision not to intubate despite an existing intubation criterion did not necessarily worsen the outcome. In this context, the current literature by von Elm et al. and Emami et al. even critically questions in principle whether intubation in severe TBI, for example, improves outcomes [36, 37]. Both conclude that there is insufficient evidence to date to support the absolute benefit of intubation in TBI. In current practice, therefore, the infrequent use of severely injured children seems to indicate that intubation is most appropriate only after all alternative treatments have been exhausted. A possible explanation for why children and adults were not intubated emerges from consideration of the multivariate regression analysis. Of all the intubation criteria, prehospital low systolic blood pressure < 90 mmHg and associated hemorrhagic shock, although significant, still emerged as the weakest parameters leading to intubation. Unlike severe TBI with a GCS < 9, alternative treatment to intubation and ventilatory therapy for hemorrhagic shock seem at least conceivable. Hudson et al. also concluded in their review that uncritical prehospital intubation for hemorrhagic shock should be reconsidered and alternative therapies should be considered [38]. In their study, Chou et al. even observed an increase in mortality with prehospital intubation due to hemorrhagic shock [39]. However, it remains completely undisputed that intubation is also of substantial importance in hemorrhagic shock to ensure adequate oxygenation. Furthermore, the most severe injuries are often associated with TBI and a GCS score < 9. In these cases, according to current studies, intubation should be performed anyway [34]. Another reason why low systolic pressure has not been used as the most important parameter for intubation in children could be due to physiologically low blood pressure values, especially in infants. However, it was shown in the present study that, for example, in Group 1 of the smallest children or babies, the prehospital blood pressure tended to be even higher on average than in the slightly older children in Group 2. Another argument against an explanation based purely on the physiology of children due to physiologically low blood pressure values is that prehospital hemorrhagic shock with a systolic pressure < 90 mmHg was also the weakest independent parameter for intubation in adults.

Overall, the respective specialization of the physician on site could also have a fundamental influence on prehospital measures. Gaessler et al. came to a similar conclusion in their study [40]. The collective here referred to patients who were transported by helicopter, regardless of whether they were children or adults. However, this relationship cannot be fully elucidated with a retrospective analysis because data on individual decision-making are simply lacking. Nevertheless, because the basic decision to intubate or not intubate was similarly distributed between children and adults in the present study, the inference that children were intubated less or more prehospital is not supported by the data presented here. As a therapy for respiratory failure, endotracheal intubation seems to be preferred over an alternative airway in both children and adults. Why this relationship exists cannot be conclusively determined based on retrospective data. It is surprising only insofar as an alternative airway would have been expected, especially in very young children, for example, out of concern that endotracheal intubation would damage vulnerable airways. However, it is also possible that the use of, e.g., a laryngeal mask as an alternative airway, is not sufficiently widespread and therefore not practiced often enough by emergency teams. This is remarkable, as the current S2k guideline Polytrauma in Children and Adolescents points out that tracheal intubation as a standard airway management procedure must be critically reflected in the pre-hospital context [9].

In summary, guidelines are an important pillar in the treatment of severely injured patients, regardless of whether they are children or adults. For example, a study by van Rein et al. demonstrated that the existence of triage protocols or checklists simplifies decision-making in the field, potentially improving outcomes [41]. A basis for such protocols should always be scientific guidelines.

### Limitations

1. In a retrospective analysis based on pseudonymized data, it is not possible to clarify the individual decisions of the hospital teams involved. Additionally, access to the patient records for further analysis was not possible due to pseudonymization.
2. Only possible links with conclusions can be described in the examined data, not absolute causalities.
3. Only patients who could be transferred to a hospital/emergency room are included in the TR-DGU. Patients who died at the scene of the accident are not documented and therefore were not evaluated.
4. All patients were treated on site by a physician. However, it remains unclear in this analysis what specialization the physician had (e.g., anesthesiologist, surgeon, etc.).
5. Particularly in children, the determination of circulatory parameters, for example, can falsify values through the use of blood pressure cuffs that do not correspond to the size. Psychological states such as anxiousness or agitation can also alter the values measured, for example, respiratory rate. However, due to the group size, these influencing factors should not have a statistical impact.

## Conclusions

The prehospital intubation criteria listed in the existing German S3 polytrauma guidelines are also applied in a pediatric collective. Based on the present results, the intubation criteria of the S3 guideline seem to be suitable, at least in practical implementation, also in children and, when applied, do not negatively influence the outcome. Although there are differences, for example, in the causes of accidents and types of injuries between adults and children; and there are only marginal differences in the implementation of prehospital intubation. Interestingly, this intubation is overtly endotracheal in children. Alternative airways are even significantly fewer compared with adults.

Although severely injured children still represent a minority of all injured patients, future guidelines should focus more on them and address them in a specialized manner. In doing so, the physiology of children must be considered. Of course, further prospective, randomized studies are needed to further investigate the issues studied here with a higher level of evidence and, in particular, to better understand the individual decisions made in the field.

## Data Availability

All data generated or analyzed during this study are included in this published article.

## Abbreviations

ACCP-SCCM: American College of Chest Physicians/Society of Critical Care Medicine
AIS: Abbreviated Injury Scale
ATLS: Advanced Trauma Life Support®
AUC: Academy for Trauma Surgery
BP: Blood pressure
CPR: Cardiopulmonary resuscitation
DGU: German Association for Trauma Surgery
GCS: Glasgow Coma Scale
ICU: Intensive Care Unit
INR: International Normalized Ratio
ISS: Injury Severity Score
MAIS: Maximum Abbreviated Injury Scale
MOF: Multiple organ failure
MV: Mean Value
NIS: Committee on Emergency Medicine, Intensive Care and Trauma Management
NISS: New Injury Severity Score
OR: odds ratio
RISC II: Revised Injury Severity Classification II
SD: Standard Deviation
SOFA: Sequential Organ Failure Assessment
SPSS®: Statistical Package for the Social Sciences
TR-DGU: TraumaRegister DGU®
TBI: Traumatic Brain Injury
